# 9-[(2-Meth­oxy­benz­yl)amino]-5-(3,4,5-trimeth­oxy­phen­yl)-5,5a,8a,9-tetra­hydro­furo[3′,4′:6,7]naphtho­[2,3-*d*][1,3]dioxol-6(8*H*)-one

**DOI:** 10.1107/S160053681202572X

**Published:** 2012-06-13

**Authors:** Shaoyu Shi, Danli Tian, Gang Luo, Ting Ai, Hong Chen

**Affiliations:** a School of Pharmaceutical Sciences, Tianjin Medical University, Tianjin 300071, People’s Republic of China; b Room of Pharmacognosy, Medical College of Chinese People’s Armed Police Forces, Tianjin 300162, People’s Republic of China

## Abstract

In the title compound, C_30_H_31_NO_8_, the tetra­hydro­furan ring and the six-membered ring fused to it both display envelope conformations, both having the same C atom as the flap. The dihedral angles between the benzene ring of the benzo[*d*][1,3]dioxole ring system and the other two benzene rings are 53.73 (3) and 83.30 (2)°. An intra­molecular N—H⋯O hydrogen bond is present. In the crystal, weak inter­molecular C—H⋯O hydrogen bonds link the mol­ecules into chains parallel to the *c* axis.

## Related literature

For the crystal structures of related podophyllotoxin derivatives, see: Luo *et al.* (2011 ▶); Li *et al.* (2011 ▶).
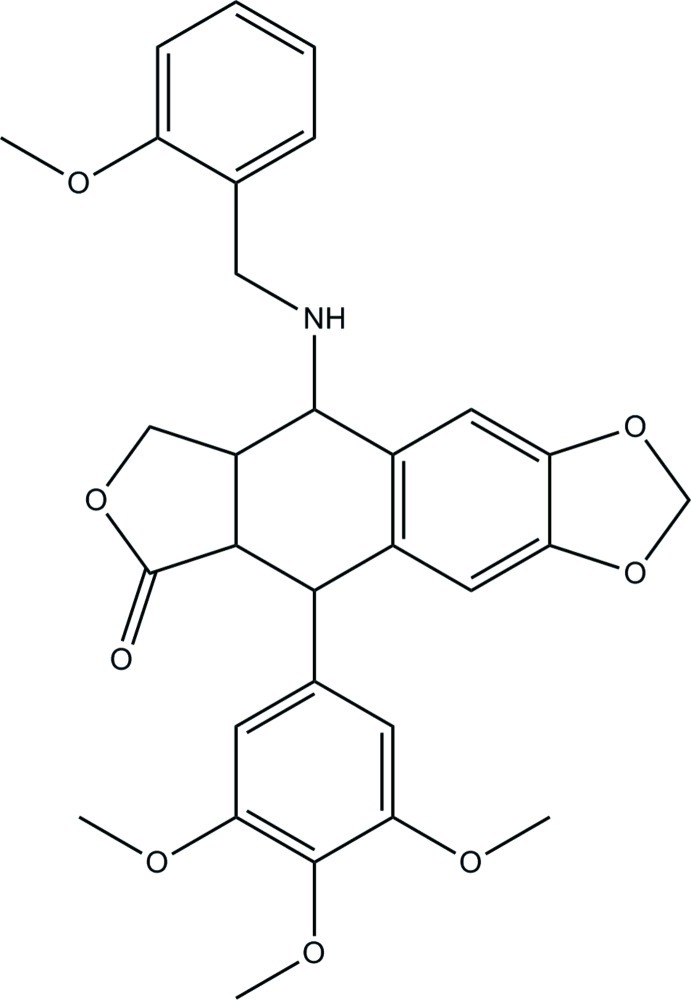


## Experimental

### 

#### Crystal data

C_30_H_31_NO_8_
*M* *_r_* = 533.56
Orthorhombic, 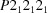

*a* = 9.6203 (19) Å
*b* = 12.870 (3) Å
*c* = 21.227 (4) Å
*V* = 2628.1 (9) Å^3^
*Z* = 4
Mo *K*α radiation
μ = 0.10 mm^−1^
*T* = 293 K
0.30 × 0.20 × 0.10 mm

#### Data collection

Rigaku Saturn diffractometer
Absorption correction: multi-scan (*CrystalClear*; Rigaku, 2007 ▶) *T*_min_ = 0.971, *T*_max_ = 0.990
22214 measured reflections
2641 independent reflections
1618 reflections with *I* > 2σ(*I*)
*R*_int_ = 0.088

#### Refinement

*R*[*F* ^2^ > 2σ(*F* ^2^)] = 0.050
*wR*(*F* ^2^) = 0.114
*S* = 0.89
2641 reflections
361 parameters
1 restraint
H atoms treated by a mixture of independent and constrained refinement
Δρ_max_ = 0.23 e Å^−3^
Δρ_min_ = −0.26 e Å^−3^

### 

Data collection: *CrystalClear* (Rigaku, 2007 ▶); cell refinement: *CrystalClear*; data reduction: *CrystalClear*; program(s) used to solve structure: *SHELXS97* (Sheldrick, 2008 ▶); program(s) used to refine structure: *SHELXL97* (Sheldrick, 2008 ▶); molecular graphics: *SHELXTL* (Sheldrick, 2008 ▶); software used to prepare material for publication: *SHELXTL*.

## Supplementary Material

Crystal structure: contains datablock(s) I, global. DOI: 10.1107/S160053681202572X/rz2767sup1.cif

Structure factors: contains datablock(s) I. DOI: 10.1107/S160053681202572X/rz2767Isup2.hkl

Additional supplementary materials:  crystallographic information; 3D view; checkCIF report

## Figures and Tables

**Table 1:** Hydrogen-bond geometry (Å, °)

*D*—H⋯*A*	*D*—H	H⋯*A*	*D*⋯*A*	*D*—H⋯*A*
N1—H1⋯O1	0.91 (1)	2.28 (3)	2.947 (5)	130 (3)
C3—H3⋯O4^i^	0.93	2.50	3.321 (6)	147

Symmetry code: (i) 
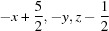
.

**Fig. 1.**
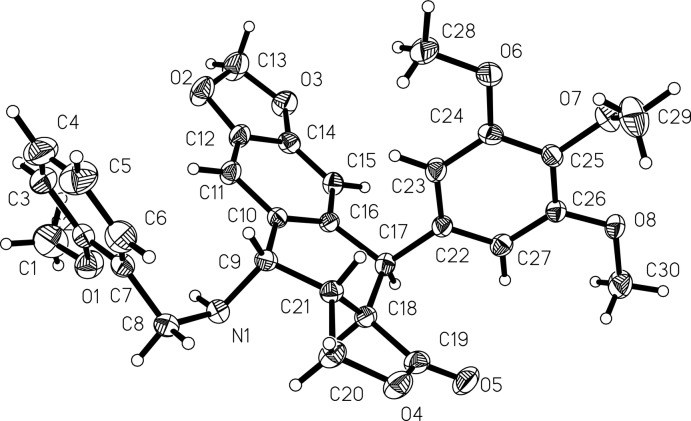
View of the title compound, with displacement ellipsoids drawn at the 30% probability level.

## supplementary crystallographic information

### Comment

Podophyllotoxin is well known for its antitumor activity. Based on the structure-activity relationships of podophyllotoxins and in order to find compounds with superior bioactivity and overcoming multidrug resistance, in continuation of our structural study of new derivatives of podophyllotoxin (Luo *et al.*, 2011; Li *et al.*, 2011), we report here the crystal structure of title compound.

In title compound (Fig. 1), bond lengths and angles are normal and in good agreement with those reported previously for related compounds (Luo *et al.*, 2011; Li *et al.*, 2011). The tetrahydrofuran ring (C18—C21/O4) and the six-membered ring (C9—C10/C16—C18/C21) fused to it both display envelope conformations, with atom C21 displaced by 0.590 (4) and 0.614 (4) Å from the mean planes through O4/C18–C20 and C9/C10/C16–C18, respectively. The dihedral angles between the benzene ring (C10—C16) of the benzo[*d*]-[1,3]dioxole ring system and the other two benzene rings (C2—C7 and C22—C27) are 53.73 (3) and 83.30 (2) °, respectively. The molecular conformation is enforced by an intramolecular N—H···O hydrogen bond (Table 1). There are weaker C—H···O intermolecular interactions, linking molecules into chains parallel to [0 0 1].

### Experimental

4β-Aminopodophyllotoxin (1.0 mmol), 2-methoxybenaldehyde (1.0 mmol) and ethylic acid (0.01 mmol) were dissolved in 95% ethanol. The mixture was stirred at room temperature for 6 h. The residue was purified by column chromatography on silica gel using petroleum ether/ethyl acetate (1:1 *v*/*v*) as eluent. Then the obtained compound (1 mmol) and NaBH_4_ (4 mmol) were dissolved in dry methanol. The mixture was stirred at 0°C for 3 h. The residue was purified by column chromatography on silica gel using petroleum ether/ethyl acetate (1:1 *v*/*v*) as eluent. Single crystals suitable for X-ray diffraction were prepared by evaporation of the solution at room temperature.

### Refinement

The amine H atom was located in a difference Fourier map and refined isotropically with the N—H distance constrained to be 0.91 (1) Å. All other H atoms were found on difference maps, and included in the final cycles of refinement using a riding model, with C—H = 0.93–0.98 Å and with *U*_iso_(H) = 1.2*U*_eq_(C) for aryl and methylene H atoms and 1.5*U*_eq_(C) for the methyl H atoms. In the absence of significant anomalous scatterers in the molecule, 877 Friedel pairs were merged before the final refinement.

### Crystal data

**Table:**

C_30_H_31_NO_8_	*F*(000) = 1128
*M**_r_* = 533.56	*D*_x_ = 1.349 Mg m^−^^3^
Orthorhombic, *P*2_1_2_1_2_1_	Mo *K*α radiation, λ = 0.71073 Å
Hall symbol: P 2ac 2ab	Cell parameters from 6120 reflections
*a* = 9.6203 (19) Å	θ = 2.5–28.0°
*b* = 12.870 (3) Å	µ = 0.10 mm^−^^1^
*c* = 21.227 (4) Å	*T* = 293 K
*V* = 2628.1 (9) Å^3^	Prism, colorless
*Z* = 4	0.30 × 0.20 × 0.10 mm

### Data collection

**Table:**

Rigaku Saturn diffractometer	2641 independent reflections
Radiation source: rotating anode	1618 reflections with *I* > 2σ(*I*)
Confocal monochromator	*R*_int_ = 0.088
ω scans	θ_max_ = 25.0°, θ_min_ = 2.5°
Absorption correction: multi-scan (*CrystalClear*; Rigaku, 2007)	*h* = −11→11
*T*_min_ = 0.971, *T*_max_ = 0.990	*k* = −15→15
22214 measured reflections	*l* = −25→25

### Refinement

**Table:**

Refinement on *F*^2^	Secondary atom site location: difference Fourier map
Least-squares matrix: full	Hydrogen site location: inferred from neighbouring sites
*R*[*F*^2^ > 2σ(*F*^2^)] = 0.050	H atoms treated by a mixture of independent and constrained refinement
*wR*(*F*^2^) = 0.114	*w* = 1/[σ^2^(*F*_o_^2^) + (0.0574*P*)^2^] where *P* = (*F*_o_^2^ + 2*F*_c_^2^)/3
*S* = 0.89	(Δ/σ)_max_ < 0.001
2641 reflections	Δρ_max_ = 0.23 e Å^−^^3^
361 parameters	Δρ_min_ = −0.26 e Å^−^^3^
1 restraint	Extinction correction: *SHELXL*, Fc^*^=kFc[1+0.001xFc^2^λ^3^/sin(2θ)]^-1/4^
Primary atom site location: structure-invariant direct methods	Extinction coefficient: 0.0157 (16)

### Special details

**Table:**

Geometry. All e.s.d.'s (except the e.s.d. in the dihedral angle between two l.s. planes) are estimated using the full covariance matrix. The cell e.s.d.'s are taken into account individually in the estimation of e.s.d.'s in distances, angles and torsion angles; correlations between e.s.d.'s in cell parameters are only used when they are defined by crystal symmetry. An approximate (isotropic) treatment of cell e.s.d.'s is used for estimating e.s.d.'s involving l.s. planes.
Refinement. Refinement of *F*^2^ against ALL reflections. The weighted *R*-factor *wR* and goodness of fit *S* are based on *F*^2^, conventional *R*-factors *R* are based on *F*, with *F* set to zero for negative *F*^2^. The threshold expression of *F*^2^ > σ(*F*^2^) is used only for calculating *R*-factors(gt) *etc*. and is not relevant to the choice of reflections for refinement. *R*-factors based on *F*^2^ are statistically about twice as large as those based on *F*, and *R*- factors based on ALL data will be even larger.

### Fractional atomic coordinates and isotropic or equivalent isotropic displacement parameters (Å^2^)

**Table:**

	*x*	*y*	*z*	*U*_iso_*/*U*_eq_	
O1	1.3087 (4)	0.1036 (3)	0.80717 (14)	0.0614 (9)	
O2	0.8443 (3)	0.0045 (3)	0.74461 (14)	0.0665 (10)	
O3	0.6445 (3)	0.0879 (3)	0.77780 (14)	0.0571 (9)	
O4	1.0753 (4)	0.0957 (3)	1.12178 (13)	0.0585 (9)	
O5	0.8792 (4)	0.1874 (3)	1.13695 (14)	0.0635 (10)	
O6	0.5983 (3)	−0.2184 (2)	1.02072 (14)	0.0537 (8)	
O7	0.4290 (3)	−0.1483 (2)	1.11232 (14)	0.0531 (8)	
O8	0.4348 (3)	0.0507 (2)	1.14883 (12)	0.0443 (8)	
N1	1.1935 (4)	0.1096 (3)	0.93580 (16)	0.0435 (9)	
C1	1.3003 (7)	0.1341 (5)	0.7425 (2)	0.0874 (19)	
H1A	1.3867	0.1190	0.7219	0.131*	
H1B	1.2819	0.2073	0.7400	0.131*	
H1C	1.2266	0.0965	0.7223	0.131*	
C2	1.3413 (4)	0.0025 (4)	0.8201 (2)	0.0456 (11)	
C3	1.3595 (5)	−0.0736 (4)	0.7748 (2)	0.0631 (14)	
H3	1.3507	−0.0577	0.7323	0.076*	
C4	1.3908 (6)	−0.1730 (5)	0.7937 (3)	0.0794 (18)	
H4	1.4017	−0.2248	0.7635	0.095*	
C5	1.4062 (6)	−0.1971 (4)	0.8552 (3)	0.0763 (17)	
H5	1.4290	−0.2644	0.8674	0.092*	
C6	1.3874 (5)	−0.1201 (4)	0.8997 (2)	0.0609 (14)	
H6	1.3970	−0.1369	0.9421	0.073*	
C7	1.3549 (4)	−0.0195 (3)	0.8835 (2)	0.0433 (11)	
C8	1.3346 (4)	0.0647 (4)	0.93241 (19)	0.0481 (12)	
H8A	1.3578	0.0362	0.9734	0.058*	
H8B	1.3999	0.1203	0.9237	0.058*	
C9	1.0832 (4)	0.0336 (3)	0.94942 (18)	0.0382 (10)	
H9	1.1187	−0.0363	0.9407	0.046*	
C10	0.9577 (4)	0.0532 (3)	0.90818 (17)	0.0332 (9)	
C11	0.9684 (4)	0.0190 (3)	0.84537 (18)	0.0425 (11)	
H11	1.0492	−0.0123	0.8306	0.051*	
C12	0.8573 (4)	0.0331 (4)	0.80700 (19)	0.0457 (11)	
C13	0.7038 (5)	0.0262 (5)	0.7288 (2)	0.0695 (16)	
H13A	0.6522	−0.0381	0.7243	0.083*	
H13B	0.6996	0.0634	0.6891	0.083*	
C14	0.7386 (4)	0.0824 (3)	0.82683 (19)	0.0397 (10)	
C15	0.7246 (4)	0.1178 (3)	0.88648 (18)	0.0389 (10)	
H15	0.6439	0.1515	0.8993	0.047*	
C16	0.8360 (4)	0.1020 (3)	0.92869 (17)	0.0333 (9)	
C17	0.8108 (4)	0.1312 (3)	0.99722 (17)	0.0373 (10)	
H17	0.7701	0.2010	0.9984	0.045*	
C18	0.9513 (4)	0.1347 (3)	1.03028 (17)	0.0382 (10)	
H18	1.0016	0.1944	1.0129	0.046*	
C19	0.9569 (5)	0.1447 (3)	1.1010 (2)	0.0493 (12)	
C20	1.1526 (5)	0.0526 (4)	1.06892 (19)	0.0508 (12)	
H20A	1.1940	−0.0137	1.0798	0.061*	
H20B	1.2250	0.0998	1.0551	0.061*	
C21	1.0407 (4)	0.0397 (3)	1.01841 (17)	0.0380 (10)	
H21	0.9859	−0.0222	1.0285	0.046*	
C22	0.7088 (4)	0.0558 (3)	1.02781 (18)	0.0376 (10)	
C23	0.7037 (4)	−0.0481 (3)	1.00894 (19)	0.0425 (10)	
H23	0.7621	−0.0718	0.9771	0.051*	
C24	0.6120 (5)	−0.1156 (3)	1.03751 (19)	0.0415 (11)	
C25	0.5238 (4)	−0.0815 (3)	1.08588 (18)	0.0395 (10)	
C26	0.5260 (4)	0.0226 (3)	1.10325 (18)	0.0380 (10)	
C27	0.6187 (4)	0.0907 (3)	1.07381 (18)	0.0408 (10)	
H27	0.6197	0.1604	1.0854	0.049*	
C28	0.6889 (5)	−0.2543 (3)	0.9721 (2)	0.0652 (15)	
H28A	0.6730	−0.2146	0.9344	0.098*	
H28B	0.6709	−0.3264	0.9638	0.098*	
H28C	0.7837	−0.2459	0.9852	0.098*	
C29	0.4876 (6)	−0.2235 (4)	1.1542 (3)	0.0791 (18)	
H29A	0.5529	−0.2659	1.1316	0.119*	
H29B	0.4149	−0.2665	1.1709	0.119*	
H29C	0.5343	−0.1886	1.1881	0.119*	
C30	0.4447 (5)	0.1544 (4)	1.1727 (2)	0.0624 (14)	
H30A	0.5374	0.1668	1.1877	0.094*	
H30B	0.3800	0.1632	1.2068	0.094*	
H30C	0.4233	0.2029	1.1397	0.094*	
H1	1.181 (4)	0.134 (3)	0.8961 (8)	0.034 (11)*	

### Atomic displacement parameters (Å^2^)

**Table:**

	*U*^11^	*U*^22^	*U*^33^	*U*^12^	*U*^13^	*U*^23^
O1	0.074 (2)	0.065 (2)	0.0459 (18)	−0.0055 (19)	−0.0023 (17)	0.0037 (17)
O2	0.051 (2)	0.107 (3)	0.0417 (19)	0.0120 (19)	−0.0044 (15)	−0.0175 (18)
O3	0.0455 (19)	0.078 (2)	0.0483 (17)	0.0075 (16)	−0.0163 (16)	−0.0041 (17)
O4	0.068 (2)	0.069 (2)	0.0390 (17)	0.0026 (18)	−0.0069 (16)	−0.0055 (16)
O5	0.082 (3)	0.063 (2)	0.0452 (19)	0.003 (2)	0.0101 (18)	−0.0183 (17)
O6	0.063 (2)	0.0339 (17)	0.065 (2)	−0.0061 (15)	0.0148 (18)	−0.0082 (15)
O7	0.047 (2)	0.0513 (19)	0.0613 (19)	−0.0114 (16)	0.0073 (16)	0.0073 (16)
O8	0.0394 (18)	0.0526 (19)	0.0410 (16)	0.0017 (15)	0.0101 (14)	−0.0049 (14)
N1	0.042 (2)	0.044 (2)	0.045 (2)	−0.0039 (18)	0.0038 (18)	−0.0020 (19)
C1	0.094 (5)	0.108 (5)	0.060 (3)	−0.016 (4)	−0.017 (3)	0.025 (3)
C2	0.034 (3)	0.055 (3)	0.048 (3)	−0.008 (2)	0.004 (2)	−0.006 (2)
C3	0.057 (3)	0.077 (4)	0.055 (3)	−0.005 (3)	0.000 (3)	−0.020 (3)
C4	0.066 (4)	0.074 (4)	0.098 (5)	0.005 (3)	−0.006 (4)	−0.041 (4)
C5	0.082 (4)	0.056 (3)	0.090 (4)	0.021 (3)	−0.005 (4)	−0.015 (3)
C6	0.049 (3)	0.068 (4)	0.065 (3)	0.007 (3)	−0.007 (3)	0.000 (3)
C7	0.034 (2)	0.055 (3)	0.042 (2)	−0.003 (2)	−0.003 (2)	−0.005 (2)
C8	0.036 (3)	0.063 (3)	0.045 (2)	−0.007 (2)	−0.004 (2)	−0.011 (2)
C9	0.034 (2)	0.036 (2)	0.045 (2)	−0.0021 (19)	−0.0015 (19)	−0.007 (2)
C10	0.031 (2)	0.034 (2)	0.035 (2)	−0.0017 (19)	0.0006 (18)	0.0000 (18)
C11	0.032 (2)	0.059 (3)	0.036 (2)	0.005 (2)	−0.002 (2)	−0.009 (2)
C12	0.042 (3)	0.060 (3)	0.035 (2)	0.000 (2)	0.001 (2)	−0.006 (2)
C13	0.051 (3)	0.110 (5)	0.047 (3)	0.007 (3)	−0.013 (3)	−0.014 (3)
C14	0.034 (2)	0.043 (3)	0.042 (2)	−0.001 (2)	−0.008 (2)	0.003 (2)
C15	0.035 (2)	0.041 (2)	0.041 (2)	0.0063 (19)	0.000 (2)	0.002 (2)
C16	0.039 (2)	0.029 (2)	0.032 (2)	−0.0042 (19)	0.0016 (19)	0.0005 (18)
C17	0.043 (3)	0.031 (2)	0.038 (2)	0.0038 (19)	0.0060 (19)	0.0030 (18)
C18	0.047 (3)	0.032 (2)	0.036 (2)	−0.005 (2)	0.003 (2)	0.0012 (19)
C19	0.063 (3)	0.040 (3)	0.045 (3)	−0.013 (2)	0.000 (3)	−0.005 (2)
C20	0.057 (3)	0.055 (3)	0.040 (2)	0.002 (2)	−0.002 (2)	−0.003 (2)
C21	0.044 (2)	0.036 (2)	0.034 (2)	−0.0007 (19)	−0.003 (2)	0.002 (2)
C22	0.041 (2)	0.033 (2)	0.038 (2)	−0.0031 (19)	0.003 (2)	−0.003 (2)
C23	0.047 (3)	0.036 (2)	0.045 (2)	0.002 (2)	0.012 (2)	−0.002 (2)
C24	0.042 (3)	0.032 (2)	0.051 (3)	−0.005 (2)	−0.005 (2)	−0.001 (2)
C25	0.035 (2)	0.043 (3)	0.040 (2)	−0.006 (2)	0.000 (2)	0.002 (2)
C26	0.035 (2)	0.043 (2)	0.036 (2)	0.000 (2)	0.003 (2)	−0.002 (2)
C27	0.044 (3)	0.035 (2)	0.043 (2)	0.004 (2)	0.001 (2)	−0.002 (2)
C28	0.086 (4)	0.037 (3)	0.073 (4)	−0.004 (3)	0.020 (3)	−0.017 (2)
C29	0.085 (4)	0.065 (4)	0.088 (4)	0.004 (3)	0.018 (4)	0.029 (3)
C30	0.068 (4)	0.052 (3)	0.067 (3)	0.001 (3)	0.020 (3)	−0.009 (3)

### Geometric parameters (Å, º)

**Table:**

O1—C2	1.367 (5)	C10—C11	1.408 (5)
O1—C1	1.429 (5)	C11—C12	1.356 (5)
O2—C12	1.381 (5)	C11—H11	0.9300
O2—C13	1.421 (5)	C12—C14	1.373 (6)
O3—C14	1.381 (5)	C13—H13A	0.9700
O3—C13	1.427 (5)	C13—H13B	0.9700
O4—C19	1.375 (6)	C14—C15	1.352 (5)
O4—C20	1.456 (5)	C15—C16	1.412 (5)
O5—C19	1.200 (5)	C15—H15	0.9300
O6—C24	1.378 (4)	C16—C17	1.522 (5)
O6—C28	1.428 (5)	C17—C18	1.524 (5)
O7—C25	1.372 (5)	C17—C22	1.525 (5)
O7—C29	1.429 (5)	C17—H17	0.9800
O8—C26	1.355 (4)	C18—C19	1.509 (5)
O8—C30	1.431 (5)	C18—C21	1.516 (5)
N1—C9	1.471 (5)	C18—H18	0.9800
N1—C8	1.477 (5)	C20—C21	1.528 (5)
N1—H1	0.907 (10)	C20—H20A	0.9700
C1—H1A	0.9600	C20—H20B	0.9700
C1—H1B	0.9600	C21—H21	0.9800
C1—H1C	0.9600	C22—C27	1.381 (6)
C2—C7	1.382 (6)	C22—C23	1.397 (5)
C2—C3	1.383 (6)	C23—C24	1.378 (6)
C3—C4	1.375 (7)	C23—H23	0.9300
C3—H3	0.9300	C24—C25	1.402 (5)
C4—C5	1.351 (7)	C25—C26	1.390 (5)
C4—H4	0.9300	C26—C27	1.398 (6)
C5—C6	1.380 (7)	C27—H27	0.9300
C5—H5	0.9300	C28—H28A	0.9600
C6—C7	1.375 (6)	C28—H28B	0.9600
C6—H6	0.9300	C28—H28C	0.9600
C7—C8	1.512 (6)	C29—H29A	0.9600
C8—H8A	0.9700	C29—H29B	0.9600
C8—H8B	0.9700	C29—H29C	0.9600
C9—C10	1.513 (5)	C30—H30A	0.9600
C9—C21	1.522 (5)	C30—H30B	0.9600
C9—H9	0.9800	C30—H30C	0.9600
C10—C16	1.398 (5)		
			
C2—O1—C1	117.9 (4)	C10—C16—C15	120.2 (3)
C12—O2—C13	105.1 (3)	C10—C16—C17	122.8 (3)
C14—O3—C13	105.0 (3)	C15—C16—C17	116.7 (3)
C19—O4—C20	110.6 (3)	C16—C17—C18	107.8 (3)
C24—O6—C28	116.1 (3)	C16—C17—C22	110.6 (3)
C25—O7—C29	114.6 (3)	C18—C17—C22	113.2 (3)
C26—O8—C30	117.3 (3)	C16—C17—H17	108.4
C9—N1—C8	114.4 (3)	C18—C17—H17	108.4
C9—N1—H1	109 (2)	C22—C17—H17	108.4
C8—N1—H1	102 (2)	C19—C18—C21	102.4 (3)
O1—C1—H1A	109.5	C19—C18—C17	119.5 (4)
O1—C1—H1B	109.5	C21—C18—C17	113.8 (3)
H1A—C1—H1B	109.5	C19—C18—H18	106.8
O1—C1—H1C	109.5	C21—C18—H18	106.8
H1A—C1—H1C	109.5	C17—C18—H18	106.8
H1B—C1—H1C	109.5	O5—C19—O4	121.5 (4)
O1—C2—C7	114.3 (4)	O5—C19—C18	130.5 (5)
O1—C2—C3	124.3 (4)	O4—C19—C18	108.0 (4)
C7—C2—C3	121.4 (5)	O4—C20—C21	102.9 (3)
C4—C3—C2	119.0 (5)	O4—C20—H20A	111.2
C4—C3—H3	120.5	C21—C20—H20A	111.2
C2—C3—H3	120.5	O4—C20—H20B	111.2
C5—C4—C3	121.2 (5)	C21—C20—H20B	111.2
C5—C4—H4	119.4	H20A—C20—H20B	109.1
C3—C4—H4	119.4	C18—C21—C9	110.7 (3)
C4—C5—C6	118.9 (5)	C18—C21—C20	101.3 (3)
C4—C5—H5	120.6	C9—C21—C20	119.4 (3)
C6—C5—H5	120.6	C18—C21—H21	108.3
C7—C6—C5	122.3 (5)	C9—C21—H21	108.3
C7—C6—H6	118.8	C20—C21—H21	108.3
C5—C6—H6	118.8	C27—C22—C23	119.5 (4)
C6—C7—C2	117.2 (4)	C27—C22—C17	119.8 (4)
C6—C7—C8	122.2 (4)	C23—C22—C17	120.7 (4)
C2—C7—C8	120.6 (4)	C24—C23—C22	120.0 (4)
N1—C8—C7	115.6 (3)	C24—C23—H23	120.0
N1—C8—H8A	108.4	C22—C23—H23	120.0
C7—C8—H8A	108.4	O6—C24—C23	123.5 (4)
N1—C8—H8B	108.4	O6—C24—C25	115.6 (4)
C7—C8—H8B	108.4	C23—C24—C25	120.9 (4)
H8A—C8—H8B	107.4	O7—C25—C26	120.3 (4)
N1—C9—C10	110.6 (3)	O7—C25—C24	120.4 (4)
N1—C9—C21	110.4 (3)	C26—C25—C24	119.1 (4)
C10—C9—C21	109.5 (3)	O8—C26—C25	115.9 (4)
N1—C9—H9	108.8	O8—C26—C27	124.4 (4)
C10—C9—H9	108.8	C25—C26—C27	119.7 (4)
C21—C9—H9	108.8	C22—C27—C26	120.8 (4)
C16—C10—C11	119.8 (4)	C22—C27—H27	119.6
C16—C10—C9	124.3 (3)	C26—C27—H27	119.6
C11—C10—C9	116.0 (3)	O6—C28—H28A	109.5
C12—C11—C10	118.0 (4)	O6—C28—H28B	109.5
C12—C11—H11	121.0	H28A—C28—H28B	109.5
C10—C11—H11	121.0	O6—C28—H28C	109.5
C11—C12—C14	122.3 (4)	H28A—C28—H28C	109.5
C11—C12—O2	127.7 (4)	H28B—C28—H28C	109.5
C14—C12—O2	110.0 (4)	O7—C29—H29A	109.5
O2—C13—O3	108.5 (4)	O7—C29—H29B	109.5
O2—C13—H13A	110.0	H29A—C29—H29B	109.5
O3—C13—H13A	110.0	O7—C29—H29C	109.5
O2—C13—H13B	110.0	H29A—C29—H29C	109.5
O3—C13—H13B	110.0	H29B—C29—H29C	109.5
H13A—C13—H13B	108.4	O8—C30—H30A	109.5
C15—C14—C12	121.7 (4)	O8—C30—H30B	109.5
C15—C14—O3	128.5 (4)	H30A—C30—H30B	109.5
C12—C14—O3	109.8 (4)	O8—C30—H30C	109.5
C14—C15—C16	118.0 (4)	H30A—C30—H30C	109.5
C14—C15—H15	121.0	H30B—C30—H30C	109.5
C16—C15—H15	121.0		
			
C1—O1—C2—C7	176.6 (4)	C16—C17—C18—C19	−170.2 (3)
C1—O1—C2—C3	−3.6 (7)	C22—C17—C18—C19	−47.5 (5)
O1—C2—C3—C4	−179.4 (5)	C16—C17—C18—C21	−49.0 (4)
C7—C2—C3—C4	0.5 (7)	C22—C17—C18—C21	73.7 (4)
C2—C3—C4—C5	−1.1 (9)	C20—O4—C19—O5	−178.7 (4)
C3—C4—C5—C6	1.1 (9)	C20—O4—C19—C18	0.3 (5)
C4—C5—C6—C7	−0.6 (9)	C21—C18—C19—O5	−157.9 (5)
C5—C6—C7—C2	0.0 (7)	C17—C18—C19—O5	−31.2 (7)
C5—C6—C7—C8	−179.9 (5)	C21—C18—C19—O4	23.3 (4)
O1—C2—C7—C6	179.9 (4)	C17—C18—C19—O4	150.0 (4)
C3—C2—C7—C6	0.0 (6)	C19—O4—C20—C21	−23.6 (5)
O1—C2—C7—C8	−0.2 (6)	C19—C18—C21—C9	−163.7 (3)
C3—C2—C7—C8	180.0 (4)	C17—C18—C21—C9	66.0 (4)
C9—N1—C8—C7	58.7 (5)	C19—C18—C21—C20	−36.0 (4)
C6—C7—C8—N1	−116.0 (5)	C17—C18—C21—C20	−166.4 (3)
C2—C7—C8—N1	64.0 (5)	N1—C9—C21—C18	75.7 (4)
C8—N1—C9—C10	−137.2 (3)	C10—C9—C21—C18	−46.3 (4)
C8—N1—C9—C21	101.5 (4)	N1—C9—C21—C20	−41.3 (5)
N1—C9—C10—C16	−102.6 (4)	C10—C9—C21—C20	−163.2 (4)
C21—C9—C10—C16	19.2 (5)	O4—C20—C21—C18	36.5 (4)
N1—C9—C10—C11	77.3 (4)	O4—C20—C21—C9	158.2 (3)
C21—C9—C10—C11	−160.8 (4)	C16—C17—C22—C27	−147.0 (4)
C16—C10—C11—C12	−1.3 (6)	C18—C17—C22—C27	91.9 (5)
C9—C10—C11—C12	178.8 (4)	C16—C17—C22—C23	31.8 (5)
C10—C11—C12—C14	2.3 (7)	C18—C17—C22—C23	−89.4 (5)
C10—C11—C12—O2	−179.0 (4)	C27—C22—C23—C24	−1.9 (6)
C13—O2—C12—C11	173.7 (5)	C17—C22—C23—C24	179.3 (4)
C13—O2—C12—C14	−7.5 (5)	C28—O6—C24—C23	1.8 (6)
C12—O2—C13—O3	12.4 (5)	C28—O6—C24—C25	−179.2 (4)
C14—O3—C13—O2	−12.6 (5)	C22—C23—C24—O6	178.5 (4)
C11—C12—C14—C15	−1.4 (7)	C22—C23—C24—C25	−0.5 (6)
O2—C12—C14—C15	179.6 (4)	C29—O7—C25—C26	−110.3 (5)
C11—C12—C14—O3	178.6 (4)	C29—O7—C25—C24	74.8 (5)
O2—C12—C14—O3	−0.4 (5)	O6—C24—C25—O7	−1.7 (5)
C13—O3—C14—C15	−172.0 (5)	C23—C24—C25—O7	177.4 (4)
C13—O3—C14—C12	8.0 (5)	O6—C24—C25—C26	−176.6 (4)
C12—C14—C15—C16	−0.5 (6)	C23—C24—C25—C26	2.5 (6)
O3—C14—C15—C16	179.5 (4)	C30—O8—C26—C25	173.3 (4)
C11—C10—C16—C15	−0.6 (6)	C30—O8—C26—C27	−6.8 (6)
C9—C10—C16—C15	179.4 (3)	O7—C25—C26—O8	2.9 (6)
C11—C10—C16—C17	173.6 (4)	C24—C25—C26—O8	177.8 (3)
C9—C10—C16—C17	−6.4 (6)	O7—C25—C26—C27	−177.0 (4)
C14—C15—C16—C10	1.5 (6)	C24—C25—C26—C27	−2.1 (6)
C14—C15—C16—C17	−173.1 (4)	C23—C22—C27—C26	2.3 (6)
C10—C16—C17—C18	19.9 (5)	C17—C22—C27—C26	−178.9 (4)
C15—C16—C17—C18	−165.7 (3)	O8—C26—C27—C22	179.8 (4)
C10—C16—C17—C22	−104.3 (4)	C25—C26—C27—C22	−0.3 (6)
C15—C16—C17—C22	70.0 (4)		

### Hydrogen-bond geometry (Å, º)

**Table:**

*D*—H···*A*	*D*—H	H···*A*	*D*···*A*	*D*—H···*A*
N1—H1···O1	0.91 (1)	2.28 (3)	2.947 (5)	130 (3)
C3—H3···O4^i^	0.93	2.50	3.321 (6)	147

Symmetry code: (i) −*x*+5/2, −*y*, *z*−1/2.
